# Glucosinolate Content in *Brassica* Genetic Resources and Their Distribution Pattern within and between Inner, Middle, and Outer Leaves

**DOI:** 10.3390/plants9111421

**Published:** 2020-10-23

**Authors:** Ju-Hee Rhee, Susanna Choi, Jae-Eun Lee, On-Sook Hur, Na-Young Ro, Ae-Jin Hwang, Ho-Cheol Ko, Yun-Jo Chung, Jae-Jong Noh, Awraris Derbie Assefa

**Affiliations:** 1 National Agrobiodiversity Center, National Institute of Agricultural Sciences, RDA, Jeonju 54874, Korea; rheehk@korea.kr (J.-H.R.); protect46284628@gmail.com (S.C.); jnlee88@korea.kr (J.-E.L.); oshur09@korea.kr (O.-S.H.); nonanona@korea.kr (N.-Y.R.); hyj6138@korea.kr (A.-J.H.); 2 Client Service Division, Planning and Coordination Bureau, RDA, Jeonju 54875, Korea; hchko@korea.kr; 3 National Creative Research Laboratory for Ca^2+^ signaling Network, Jeonbuk National University Medical School, Jeonju, 54896, Korea; yjchong@jbnu.ac.kr; 4 Jeonbuk Agricultural Research and Extension Services, Iksan 54591, Korea; nohjj@korea.kr

**Keywords:** *Brassica rapa* L., kimchi cabbage, multiple reaction monitoring, principal component analysis, glucosinolates, genetic resources

## Abstract

Glucosinolates (GSLs) are sulfur-containing secondary metabolites naturally occurring in *Brassica* species. The purpose of this study was to identify the GSLs, determine their content, and study their accumulation patterns within and between leaves of kimchi cabbage (*Brassica rapa* L.) cultivars. GSLs were analyzed using UPLC-MS/MS in negative electron-spray ionization (ESI^−^) and multiple reaction monitoring (MRM) mode. The total GSL content determined in this study ranged from 621.15 to 42434.21 μmolkg^−1^ DW. Aliphatic GSLs predominated, representing from 4.44% to 96.20% of the total GSL content among the entire samples. Glucobrassicanapin (GBN) contributed the greatest proportion while other GSLs such as glucoerucin (ERU) and glucotropaeolin (TRO) were found in relatively low concentrations. Principal component analysis (PCA) yielded three principal components (PCs) with eigenvalues ≥ 1, altogether representing 74.83% of the total variation across the entire dataset. Three kimchi cabbage (S/No. 20, 4, and 2), one leaf mustard (S/No. 26), and one turnip (S/No. 8) genetic resources were well distinguished from other samples. The GSL content varied significantly among the different positions (outer, middle, and inner) of the leaves and sections (top, middle, bottom, green/red, and white) within the leaves. In most of the samples, higher GSL content was observed in the proximal half and white sections and the middle layers of the leaves. GSLs are regarded as allelochemicals; hence, the data related to the patterns of GSLs within the leaf and between leaves at a different position could be useful to understand the defense mechanism of *Brassica* plants. The observed variability could be useful for breeders to develop *Brassica* cultivars with high GSL content or specific profiles of GSLs.

## 1. Introduction

Glucosinolates (GSLs), also called *β*-thioglucoside-*N*-hydroxysulfates, are a class of sulfur-containing important plant secondary metabolites naturally occurring in *Brassica* species [1]. GSLs are most frequently classified as aliphatic, aromatic, and indole GSLs based on the structure of their side chain (R group). The side chain is mainly derived from amino acid precursors including methionine (and also alanine, leucine, isoleucine, or valine in some cases) for aliphatic, phenylalanine for aromatic, and tryptophan for indole GSLs [2]. However, this classification wrongly used the names aliphatic, aromatic, and indole as synonyms of methionine, phenylalanine-, and tryptophan-derived GSLs, respectively [3]. Blažević et al. [3] presented a more meaningful classification alternative to that previously used: (i) based on the amino acid precursor (tryptophan-derived versus isoleucine-derived versus methionine-derived); (ii) according to the type of degradation product (stable isothiocyanate-yielding versus thiocyanate ion-yielding versus oxazolidine-2-thione yielding GSLs); and (iii) according to the presence or absence of an aromatic moiety in the GSL. Most GSLs share a basic chemical structure consisting of a *β*-D-glucopyranose residue linked via a sulfur atom to a (*Z*)-*N*-hydroximinosulfate ester and a variable R group [4]. Glucosinolates and their degradation products exhibit wide ranges of biological activities, including both negative and positive nutritional attributes and the mediation of plant–herbivore interactions. Upon hydrolysis by myrosinases, GSLs produce several degradation products, such as isothiocyanates, thiocyanates, oxazolidinthiones, epithionitriles, and nitriles [5]. GSLs and their biosynthetic products are implicated to reduce the risk of cancer in humans [6,7,8] and exhibit antimicrobial activities [9,10,11]. The health-related functions of GSLs are dictated by their bioavailability. GSLs and their degradation products undergo transformation, assimilation, absorption, and elimination after ingestion in the human gut [12,13]. Although their contribution is complex to understand, GSLs are also regarded as an important component of flavor in cooked vegetables [14]. GSLs and their degradation products mediate the process of plant defense mechanism against danger by serving as a feeding deterrent to a wide range of herbivores such as birds, mammals, mollusks, aquatic invertebrates, nematodes, bacteria, and fungi [14,15,16]. Contrary, the same GSLs attract and stimulate specialist herbivores such as the larvae of the lepidopteran species *Plutella xylostella* and *Pieris rapae* [15], which often use these compounds as cues for feeding or oviposition. The biocidal activity of GSL-containing *Brassica* plants has made them a promising alternative to synthetic pesticides for pest and disease control [17,18]. In planta studies of various *Brassica* seedlings have also shown a positive correlation between GSL content and disease severity [19]. 

GSLs are reported to be found in the vegetative and reproductive tissues of various dicotyledonous plant families and are the major secondary metabolites in mustard-oil plants of the *Brassicaceae* family [4,20]. The main food sources of glucosinolates are reviewed by Possenti et al. [21]. The content of GSLs accounts for around 1% of the dry weight in *Brassica* vegetables and can reach up to 10% in the seeds of some plants [4]. The qualitative and quantitative profiles of total and individual GSLs in *Brassica* vegetables vary significantly due to several factors such as cultivar genotype [22,23], developmental stage [24], environmental conditions (temperature, light, water, and soil) [25,26,27,28], growing seasons [29], agricultural practices [30], level of insect damage [27,31], and post-harvest conditions [32]. Wide geographic and evolutionary variation is recorded in broccoli [29], *A. thaliana* [33], Chinese cabbage [23], and cabbage (*B. oleracea* L.) [34]. Apart from the aforementioned factors, GSLs tend to vary quantitatively and qualitatively based on plant part, as observed in kale [27], in cabbage [26], and *A. thaliana* [24]. 

Commonly, GSLs are extracted using boiling water/methanol followed by desulfonation of intact GSLs on Sephadex-A25 columns [34], followed by quantitation and identification by HPLC. However, the desulfonation process has been found to be laborious and time-consuming [35], and some GSLs could be insufficiently desulfonated in the process at a lower concentration of sulfatase [36]. GC-MS methods are often used for detailed analysis [37]. Recently, a simplified method of sample extraction from lyophilized samples followed by quantitation and identification of intact GSLs using UPLC-DAD-MS/MS in multiple reaction monitoring (MRM) mode was reported [38]. 

Leaves of kimchi cabbage, turnip, mibuna, leaf mustard, and cabbage are commonly used for various dishes in many countries. Kimchi cabbage is a major ingredient in kimchi and a widely consumed traditional fermented food in Korea [23]. Several comparative studies on the profiles of GSLs in *Brassica* germplasm collections across the world are available in the literature [1,22,29,39,40,41]. However, most of the studies so far are focused on the levels of GSLs in the seeds of *Brassica* plants [9,22,42]. Lee et al. [23] identified and quantified ten different GSLs in breed varieties of kimchi cabbage collected from the Republic of Korea. Studies on diverse collections of genetic resources such as gene bank germplasm collections are elusive. Reports about the variability of GSLs on leaves of *B. rapa* L. were also given less attention compared to seeds. Yang and Quiros [43] found extensively varied GSL content among *B. rapa* L varieties. Accessions from a Russian gene data bank showed a wide variety of GSLs qualitatively and quantitatively among the genetic resources [44]. Another study on varieties of turnip greens from Spain also showed wide diversity in the quality and quantity of GSLs [45]. The wide range of variability in the type and amount of GSLs from different countries [44,45,46], in addition to other experimental related factors, could underline the variability in the GSL biosynthesis pathway within the plant to adapt the surrounding conditions. Many plant natural products, including GSLs, serve as defenses against herbivores [31]. It is important to determine the GSL content in different tissues of the plant to understand the actual defense role that a potential herbivore would encounter. In this study, we have identified and quantified eight GSLs, namely gluconapin, glucobrassicanapin, progoitrin, glucotropaeolin, glucoerucin, gluconasturtiin, glucoberteroin, and glucobrassicin, in 48 genetic resources including kimchi cabbage (*B. rapa* L.), turnip (*B. rapa* L.), mibuna (*B. rapa* L.), leaf mustard (*Brassica juncea* L. Czern.)*,* and cabbage (*B. oleracea* L.) collected from China, Ethiopia, Japan, North Korea, South Korea, and Taiwan. The crops were grown in uniform agricultural conditions. Moreover, the spatial accumulation patterns of GSLs within and between the leaves of three kimchi cabbage commercial cultivars have been determined. 

## 2. Materials and Methods

### 2.1. Reagents and Standards

All chemicals and solvents used during extraction and analysis were of analytical grade and purchased from Fisher Scientific Korea Ltd. (Seoul, South Korea) and Sigma-Aldrich (St. Louis, MO, USA). GSL standards (gluconapin, glucobrassicanapin, progoitrin, glucotropaeolin, glucoerucin, gluconasturtiin, glucoberteroin, and glucobrassicin) were purchased from Phytoplan Diehm & Neuberger GmbH (Heidelberg, Germany). All individual GSL standards had purity greater than or equal to 97%. 

### 2.2. Plant Materials

The seeds of 48 genetic resources (43 germplasm collections and five commercial cultivars), belonging to *B. rapa* L., *Brassica juncea* L. Czern., and *B. oleracea* L. and originating from six different countries (China (13), Ethiopia (1), Japan (1), North Korea (1), South Korea (12), and Taiwan (20)) were obtained from the gene bank of South Korea and grown at the research farm of the National Agrobiodiversity Center (NAC), Jeonju (35°49′18″ N 127°08′56″ E), Republic of Korea. Seeds were sown in plug trays in the last week of August, 2018, and seedlings were grown inside a greenhouse. After a month, healthy-looking seedlings (4 to 5 leaves) were transplanted to an area of 60 × 40 cm per plant in an experimental field of NAC. Harvesting was conducted in the first week of November. Plant cultural practices were followed as per the recommendation of the Rural Development Administration (RDA) of South Korea. Fertilizers (N-K-P-Ca-B = 65-45-100-100-1.5 kg/10a) were applied before transplanting the seedlings followed by RDA’s standard, and drip irrigation tape was used for watering. One teaspoon per plant of nitrogen fertilizer was applied when the plant started to form bulbs (12–14 leaves). Each accession consisted of 25 plants. Plant growth was maintained using nutrient solution throughout the growing season. As external damage could alter the content of GSLs, the plant materials were protected from any damage, and 10 to 15 healthy plants were used for sampling for the analysis of GSLs. Leaves were collected from the outer, inner, and middle location of each plant and mixed. In each accession, three replicate samples were prepared. Great care was taken to prevent thawing of the sample to minimize enzymatic degradation of GSLs. Samples were immediately frozen and all equipment in contact with them was held at subzero temperatures until further processing. 

**Figure 1:**
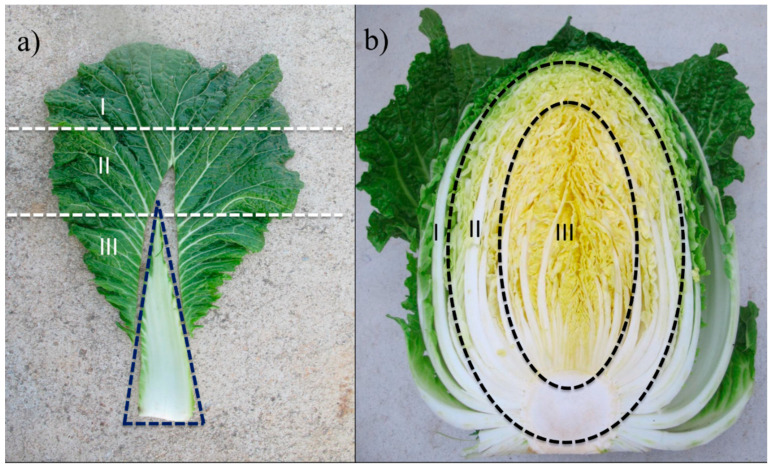
Representative photos of sampling positions of kimchi cabbage based on (**a**) leaf sections: I, III, III refers to the upper, middle, and bottom parts of the leaf. The white section is indicated by the triangular dashed line. The green/red part was sampled from the whole leaf excluding the white section. (**b**) Location of the leaves in the whole plant: I, II, and III refer to the outer (two layers), middle (three layers), and inner (the remaining) parts of the vegetable.

To study the GSL spatial distribution within sections of the leaf of kimchi cabbage and between leaves, two green-pigmented (“Hangamssam” and “Alchandul”) and one red-pigmented (“Bbalgang3-ho”) commercial cultivars were selected. The inner, middle, and outer leaves were separated. Each leaf was then dissected into the top, middle, bottom, green/red, and white parts as required. Three replicates were prepared from 15 healthy plants accordingly. Sampling positions of kimchi cabbage plants are shown in Figure 1. Additional information about the germplasm collections and commercial cultivars is presented in Table 1.

### 2.3. Sample Pretreatment, Extraction, and Analysis of GSLs

Samples were harvested, placed in a vinyl freezer bag, and kept at −80 °C until further processing. The frozen samples were subsequently lyophilized for 48 h using LP500 vacuum freeze-drier (Ilshinbiobase Co., Seoul, Korea), ground to fine powder, and kept at −80 °C until analysis. The extraction of GSLs was conducted following the method reported by Ishida et al. (2011) [47]. Briefly, 0.1 g sample was mixed with 5 mL of 80% methanol, held at 25 °C for 30 min, and shaken at 120 r/min for 30 min at room temperature. The mixture was centrifuged using VS-180CFi centrifuge (Vision Scientific Co., Daejeon, Korea) (centrifuge conditions set at 14,000 rpm, 4 °C, and 10 min). The supernatant was transferred into a vial and GSLs were analyzed immediately using UPLC-MS/MS.

Intact GSLs were analyzed using an Acquity UPLC System (Waters, Milford, MA, USA) coupled to Xevo™ TQ-S system (Waters, MS Technologies, Manchester, UK). Chromatographic separation was carried out using Acquity UPLC BEH C18 (1.7 μm, 2.1 × 100 mm) column (Waters Corp., Manchester, UK). The flow rate was kept at 0.5 mL/min; the column temperature was maintained at 35°C, and the injection volume was 5 μL. The mobile phase was composed of 0.1% trifluoroacetic acid in water as eluent A and 0.1% trifluoroacetic acid in methanol as eluent B. The elution conditions were as follows: initial condition set at 100% of A; 0.0–1.0 min, 100% of A; 1.0–7.0 min, 100 to 80% A; 7.0–10 min, 80 to 0% of A; 10–11 min, 0 to 100% of A; 11–15 min, 100% of A. The mass spectrometry instrument was operated in negative ion electrospray ionization (ESI^−^) and multiple reaction monitoring (MRM) mode. Data acquisition was performed using MassLynx 4.1 software. For MS/MS detection, the ionization source parameters were set as follows: the capillary and con voltages were set as 3kV and 54 v, respectively; the ion source and the desolvation temperatures were set as 150 and 350 °C, respectively. The cone and desolvation gas were set at flow rates of 150 and 650 Lh^−1^, respectively. GSLs were identified by comparing their retention times and MS and MS/MS fragmentation spectra with those of commercial standards. Individual GSLs were quantified by MRM, considering one MS/MS transition for each compound. Selected transitions and other MRM parameters are presented in Table 2. The final concentration of individual GSLs was calculated using linear regression equations derived from the calibration curves of the corresponding standards. Results were calculated from peak area responses and presented as µmolkg^−1^ sample dry weight (DW).

The established UPLC-MS/MS method of analysis was validated by measuring the linear, intraday, and interday precision. Standard stock solutions of glucosinolates were prepared by dissolving 10 mg in methanol to obtain a final concentration of 1 mg/mL. Standard calibration curves that were used to quantify the GSLs were prepared from serially diluted solutions (1000 to 1 ng/mL) from the stock solution. Calibration curve parameters are presented in Table 2. The precision of the method was determined as the percentage of the ratio of the standard deviation to the mean value (relative standard deviation, RSD) of interday and intraday analysis. Both precision and accuracy of the method were within the acceptable limit of ± 15% of the actual values. The limit of detection (LOD) and limit of quantification (LOQ) values were determined as, respectively, three and ten times the standard error of the intercept of the regression equation of the linear calibration curve divided by the slope. Based on the residual standard deviation of the response and the slope, the LODs for the nine GSLs ranged between 0.5 and 1 ng/mL, and LOQs were between 1.5 and 3 ng/mL. Test solutions were prepared freshly before analysis.

### 2.4. Statistical Analysis

Results were expressed as mean ± standard deviation (SD) of triplicates. The data were treated with analysis of variance (ANOVA) followed by Duncan’s multiple range test (*p* < 0.05) using the SPSS V. 17.0 statistical program (SPSS Inc., Chicago, USA). Principal component analysis (PCA) was performed using the statistical program R (Rstudio, Inc., Austria). Data were visualized using principal components score and loading plots (PCA-Biplot). Points represented an individual sample, and the lines represented the contribution of an individual GSL to the score.

## 3. Results and Discussion

In this study, eight GSLs were identified and quantified in leaves of five commercial varieties and 45 germplasm collections of *Brassica* plants belonging to *B. rapa* L., *B. juncea* L. Czern., and *B. oleracea* L. The concentrations of GSLs were also evaluated in various leaf sections and positions of two green- (“Hangamssam” and “Alchandul”) and a red- (“Bbalgang 3-ho”) pigmented commercial varieties commonly called kimchi cabbage. Five aliphatic (GNA, GBN, PRO, ERU, and BER), two phenylalkyl (TRO and NAS), and one indole (GBC) GSLs were identified. GSLs were examined using negative ionization electrospray (ESI^−^) LC-MS/MS in MRM mode by monitoring specific transitions originating the characteristic fragment ions (Table 2). The results of this study, presented and discussed in detail in the next sections, showed that the values varied widely among the entire germplasm collections and between different sections and positions of the *Brassica* leaves. Principal component analysis (PCA) was employed to identify the GSL exhibiting the greatest variance across the entire collection and to determine closely related individual GSLs.

### 3.1. Variation in GSL Content between Germplasm Collections

As can be seen in Table 3, a significant difference in GSL content was observed among the germplasm collections and commercial varieties of *Brassica* plants. The total GSL content ranged from 621.15 (“Alchandul”, S/No. 42) to 42,434.21 (IT 260822, S/No. 2) µmol kg^−1^ DW with an average value of 14,050.97 µmol kg^−1^ DW. Aliphatic GSLs were dominant throughout the entire collections, which altogether represented from 4.44% to 96.2% (average 66.12%) of the total GSL content, followed by phenylalkyl GSLs (0.90%~81.32%; average 17.56%). GBC, the only indole GSL detected in our study, represented as low as 1.36% and as high as 69.59% of the total GSLs. GBN (0.04~23,026.64 µmol kg^−1^ DW), representing an average of 45.06% was the most dominant GSL across the entire collections. GNA (11.90 ~ 15,276.50 µmol kg^−1^ DW), GBC (120.81~12,134.40 µmol kg^−1^ DW), and NAS (46.60 ~ 6353.11 µmol kg^−1^ DW), representing an average 13.47%, 16.31%, and 17.37%, respectively, represented a moderate proportion. The least dominant GSLs were BER, PRO, ERU, and TRO and presented average values of 433.35, 426.15, 52.17, and 13.46 µmol kg^−1^ DW in the entire samples, respectively. Some accessions were found to accumulate unusually high content of a particular type of GSL. For example, one turnip (S/No 8) and four kimchi cabbage genetic resources (S/No. 10, 13, 22, and 27) contained more than 90% aliphatic glucosinolates. The highest amount of GBC, the only indole GSL detected, was detected in one cabbage (S/No. 45) and two kimchi cabbage germplasm (S/No. 20 and 35). Accession 47 (IT 100409) had the highest content of phenylalkyl GSL (81.32%), where NAS being contributed most. Accessions 12 (IT 228167) and 20 (IT 32750) had the highest ERU and GBC content, respectively, accounting about 3.5-fold higher than the accessions containing the second-highest in the entire sample. 

Most of the accessions were originated from Taiwan (20), China (13), and South Korea (12). Taiwanese originated *Brassica* resources exhibited higher averaged combined GSL content (16, 392 µmol kg^−1^ DW), followed by Chinese (15, 794 µmol kg^−1^ DW) and South Korean (8,156 µmol kg^−1^ DW) originated resources. In terms of individual glucosinolates, Taiwanese originated resources had the highest GNA, GBN, TRO, and ERU, while Chinese originated materials excel in PRO, NAS, and GBC levels. South Korean originated resources were superior in their BER content. The PCA plot of the first two components showed that the genotypes were distributed throughout the four quadrants with no significant grouping based on their country of origin, suggesting the absence of intrinsic similarities between them in their GSL content based on their origin (Supplementary File, Figure S2).

GNA and GBN were documented as the most abundant GSLs in the leaves of *B. napa*, as reported previously [23,44,45,48]. However, GBN, 4-methoxyglucobrassicin, and PRO were dominant in the same crop in another study [49]. The identity and quantity of GSLs vary considerably between various crops of *Brassica*. For example, the predominant GSLs in broccoli were glucoraphanin, GNA, and GBC, while sinigrin was found to be the dominant GSL in green cabbage, Brussels sprouts, cabbage, cauliflower, and kale [22,34]. This study revealed a wide variety of GSLs among accessions of *Brassica* germplasm collections. The difference observed in the GSL profile is both qualitative and quantitative. This could determine their level of nutritional and health-promoting properties and supports the feasibility of developing cultivars with an enhanced level of GSLs through genetic manipulation. Previous studies showed the impact of temperature [27], amount of rainfall [50], radiation [51,52], plant part examined [1], phenological stage of growth [24,27], and level of insect damage [27,53] on the level of GSLs.

Other *Brassica* plant leaf sources of the GSLs investigated in our study include but are not limited to broccoli, Brussels sprout, cauliflower, kale, Chinese cabbage, rocket plants, pak choi, and watercress [48,54,55,56,57,58,59,60,61,62,63,64]. We compared the levels of GSLs in other *Brassica* plants in previous reports that employed LC/MS and LC/MS/MS methods. The most dominant GSL in this study, GBN, was previously reported in the ranges of 970 to 10,480 µmol/kg DW [46] and 400 to 8080 µmol/kg DW [48] in Chinese cabbage and from 2.52 to 20.19 µmol/kg DW in pak choi [55]. The GNA levels ranged from 250 to 11,100 µmol/kg DW [46] and 400 to 8990 µmol/kg DW [48] in Chinese cabbage, which were in agreement with our study. However, compared to our results, quite higher (4910 to 70,670 µmol/kg DW) and lower (ND to 340 µmol/kg DW) levels were recorded in pak choi [55] and rocket [63], respectively. Comparable levels of PRO were obtained in rocket (187.4 to 624.7 µmol/kg DW) [62] and Chinese cabbage (140 to 3520 µmol/kg DW) [46] to our study. Pak choi contained high PRO (1160 to 41,510 µmol/kg DW) compared to other *Brassica* plants in previous reports and this study [55]. Broccoli (379.2 to 2895.2 µmol/kg DW), Brussels sprouts (14,92.9 to 2532.6 µmol/kg DW), cauliflower (655.5 to 2887.6 µmol/kg DW) [59], kale (3200 to 7250 µmol/kg DW) [57], Chinese cabbage (130 to 6810 µmol/kg DW) [48], and pak choi (880 to 4860 µmol/kg DW) [55] contained moderately comparable levels of GBC to our samples, while rocket (17.8 to 44.6 µmol/kg DW) had a significantly lower amount [62]. Watercress had high levels of NAS (4155.8 µmol/kg DW) [56] compared to other *Brassica* plants but in corcondance with this study. ERU was recorded as a dominant GSL in rocket [58,60,61,62,63,64] and much higher compared to this study, but comparable results were obtained in pak choi (ND to 2370 µmol/kg DW) [55] and Chinese cabbage (40 to 750 µmol/kg DW) [48].

### 3.2. Intra- and Inter-Leaf Distribution of GSLs in Kimchi Cabbage

**Figure 2:**
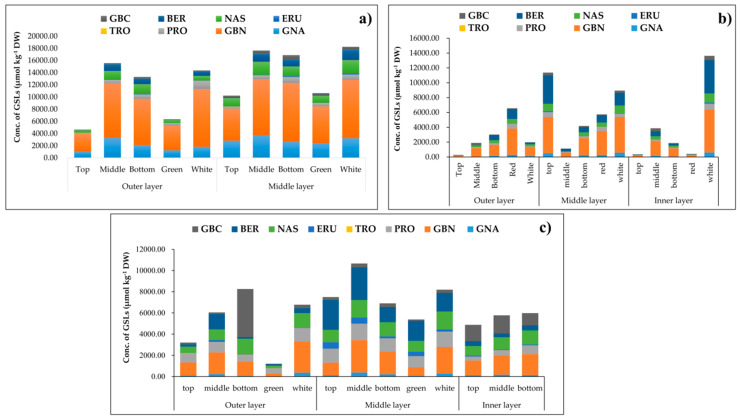
Glucosinolate levels in different leaf sections of three cultivars of kimchi cabbage: (**a**) Hangamssam; (**b**) Bbanlgang 3-ho; and (**c**) Alchandul.

The leaves of three green-/red-pigmented kimchi cabbage cultivars including “Hangamssam” (green), “Alchandul” (green), and “Bbalgang 3-ho” (red) were segregated based on their position in the whole plant as inner, middle, and outer layers. Each leaf was further portioned into different sections (top, middle, bottom, green/red, and white). The GSL content in kimchi cabbage significantly varied based on leaf section, position, and color. The GSL content in different leaf sections/positions of the three kimchi cabbage cultivars is presented in Figure 2 and Supplementary File (Table S1). The leaf parts were sampled as demonstrated in Figure 1. The white section within the leaf contained a higher total sum of GSLs (1.52 to 33.07-fold higher) than the green/red section, except in the outer layer of “Bbalgang 3-ho”, where the red section contained a 3.31-fold higher total GSL concentration than the white section. The trend in total GSL content within different leaf sections (top, middle, and bottom) was not strictly consistent. However, in most cases, higher GSL content was observed at the proximal half of the leaves. Concerning the position of the leaf (outer, middle, and inner layers) in the whole plant, the average total GSL content in the middle layers was 1.34-, 1.42-, and 3.21-fold higher than in the outer layers of “Hangmassam”, “Alchandul”, and “Bbalgang 3-ho” cultivars, respectively. The content of total GSLs evaluated in the inner layers of “Alchandul” and “Bbalgang 3-ho” showed no significant difference with the outer layers. In general, the middle layer leaves were found to contain higher concentrations of GSLs compared to older (outer) leaves and the younger inner layer leaves. The green-pigmented cultivars showed superiority in total glucosinolate content over the red-pigmented cultivars. In an earlier study, the inner layers of *B. oleracea* var. *capitate* leaves were reported to exhibit 1.1- to 1.8-fold higher GSL concentrations than the outer positions [65]. In another study, younger leaves of *Raphanus sativus* were found to contain higher GSL content [66].

The enhancement of GSL concentration upon plant damage [53] has long indicated that GSLs are plant defense chemicals where mostly their defensive properties are attributed to the toxicity and deterrence nature of their degradation products [15]. In contrast, there are also cases where GSLs mediated by their volatile hydrolysis products could serve to attract adapted herbivores that often use GSLs as cues for feeding or oviposition [15]. The spatial distribution of GSLs in different sections of a single leaf and/or location of the leaf in the whole plant could partly be important to explain the patterns of herbivory. Studies devoted to GSL spatial patterns within leaves of kimchi cabbage are elusive. The proximal halves of *R. sativus* leaves contained a higher mean concentration of GSLs compared to the distal halves of leaves [66]. Shroff et al. (2008) [67] studied the spatial distribution (midvein, inner lamina, and outer lamina) of GSLs in leaves of *A. thaliana* and tried to relate the distribution to the pattern of herbivory caused by larvae of the lepidopteran, *Helicoverpa armigera.* These authors found out that the GSL abundance in the inner vs. the peripheral part of the leaf affected insect feeding preference and anti-herbivore defenses. As stated in the previous section and shown in Figure 2, the white part (midvein) of kimchi cabbage contained relatively higher GSLs compared to the green- or red-colored part. This is consistent with *A. thaliana* leaves, where the midvein part exhibited the greatest concentration compared to the other sections of the leaf [67]. This could be due to the distribution of certain biosynthetic enzymes exclusively to vascular bundles [68], resulting in greater synthesis and storage of GSLs in the midvein (white part) of the leaf of kimchi cabbage. It could also be related to ecological significance as the midvein is critical to the function of the leaf, and the transport of water and nutrients takes place through it [69]. The greater concentration of GSL in the white part of kimchi cabbage in our study corroborates the idea of the storage of GSLs being associated with the vascular system. The higher content of GSLs in the middle (younger) leaves compared to the outer (older) leaves in this study is also in agreement with the predictions of optimal defense theory: younger leaves are more valuable as they have higher future photosynthetic potential and need a higher degree of protection from damage [70]. In addition, GSL concentration could tend to decrease in outer leaves due to the dilution of GSLs as the leaf expands [70]. 

### 3.3. Multivariate Analysis

**Figure 3:**
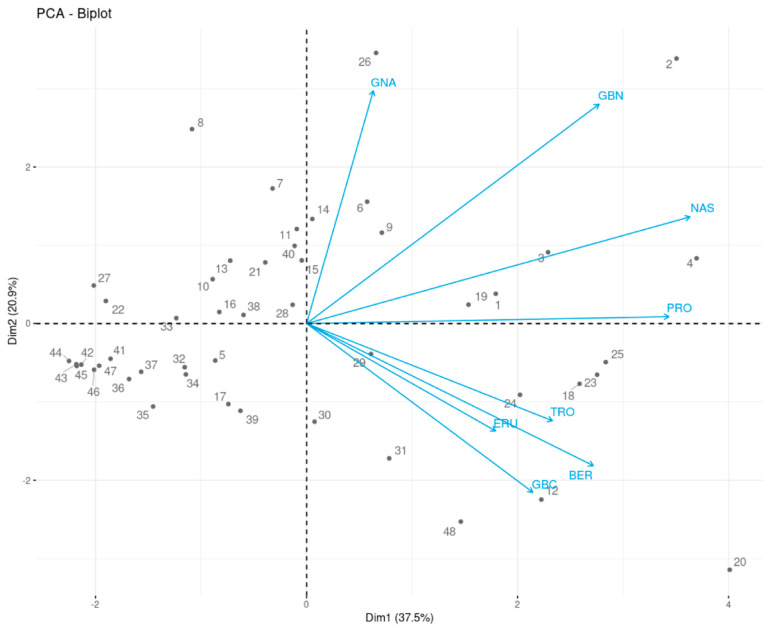
Principal component analysis (PCA) plot of the scores (indicated by dotes) and loadings (indicated by lines) of the 48 *Brassica* plants based on the first and second principal components. The numbers 1–48 correspond to the S/No in Table 1 and Table 3. GNA = gluconapin; GBN = glucobrassicanapin; PRO = progoitrin; TRO = glucotropaeolin; ERU = glucoerucin; NAS = gluconasturtiin; BER = glucoberteroin; and GBC = glucobrassicin.

The results of PCA are indicated by the principal components score and loading plots (PCA-Biplot). The PCA of GSL data yielded three principal components with eigenvalues ≥1, accounting for 74.83% of the total variance across the entire dataset. The first, second, and third principal components (PCs) contributed 37.47%, 20.88%, and 16.47% of the total variance, respectively. The loadings, eigenvalue, and percentage of variance obtained for all principal components (PCs) are presented in the Supplementary File (Table S2). Scores and loading plots of the first two PCs obtained from GSL content of 48 *Brassica* germplasm collections are presented in Figure 3. The loadings of GSLs (represented by light blue arrows) show the extent and nature of each GSL concentration contribution to the principal components. All the GSLs were positively correlated with PC1, while GNA, GBN, and NAS had a positive correlation with PC2. NAS was the predominant GSL in PC1, followed by PRO, GBN, and BER, while GNA, GBN, GBC, and BER had a major contribution to PC2, with the last two affecting it negatively. Three kimchi cabbage (S/No. 20, 4, and 2, the former located at the bottom right and the latter two at the top right quadrant of the PCA plot), one leaf mustard (S/No. 26, located at the top right quadrant of the PCA plot), and one turnip (S/No. 8, located at the top left quadrant of the PCA plot) genetic resources were seen well distinguished from other samples. The separation of S/No. 20 and S/No. 4 from other accessions in the score plot could be described by their significantly higher content of GBC and NAS, respectively. On the other hand, S/No. 2 (IT260822) had relatively high content of NAS and GBN (ranked second and third) compared to other genetic resources. S/No. 26 is characterized by its high content of GBN and GNA (ranked first and third in the entire collections, respectively) while S/No. 8 had the highest concentration of GNA in the entire collection of genetic resources. 

## 4. Conclusions

Eight GSLs were identified and quantified in *Brassica* germplasm collections and commercial varieties using the UPLC-MS/MS method in multiple reaction monitoring scan mode. Remarkable differences in total and individual GSLs were observed among different samples. The data in this study revealed a wide variation in the level of GSLs among genotypes, leaf position/section, and leaf color. The PCA in this study allowed easy visualization of the data, and five genetic resources (S/No. 20, 4, 2, 26, and 8) were seen separated from the entire collections. The inter- and intra-leaf variations of GSLs were examined in three commercial kimchi cabbage varieties. The GSL content varied significantly among leaves in different positions of the plant (outer, middle, and inner) and sections within leaves (top, middle, bottom, green/red, and white). Higher GLS content was observed in the proximal half and white sections of the leaves and middle layers in all of the samples tested. The variation in the GSL level suggests that the potential health benefits of *Brassica* plants could depend on the type of accession used. The wide variability observed in GSL content among the germplasm collections in this study offers important and basic information for enhancing the level of GSLs in *Brassica* plants through breeding and hence their health beneficial properties. Besides this, the development of *Brassica* plants with specific GSL profiles of specific health beneficial properties would help for a meaningful recommendation of dietary intake of *Brass*ica vegetables. Two aliphatic (GBN and GNA), one phenylalkyl (NAS), and one indole (GBC) were detected in relatively higher amount compared to other GSLs. As the breakdown products of these GSLs are implicated to posses antimicrobial, antibacterial, and anticancer properties elsewhere, they could be used as potential biomarkers for the consumption of kimchi cabbage. In this study, we determined the variability of GSL content and composition reflected between *Brassica* genetic resources and within and between leaves. The results would widen the present understanding of the accumulation pattern of GSLs in leaves of *Brassica* plants and provide information about the nature of plant defenses towards a perceived danger.

## Figures and Tables

**Table 1:** Accession number, scientific name, common name, and origin of 48 germplasm accessions of *Brassica* genus.

S/No	Accession No.	Scientific Name *	Crop Name	Given Name	Origin	Classification
1	IT260816	*Brassica rapa* L.	Kimchi cabbage	CHINA-YAAS-2010-103	China	Breeding line
2	IT 260822	*Brassica rapa* L.	Kimchi cabbage	CHINA-YAAS-2010-109	China	Breeding line
3	IT 100390	*Brassica rapa* L.	Kimchi cabbage	AVRDC-KJH-1985-100390	Taiwan	-
4	IT 260819	*Brassica rapa* L.	Kimchi cabbage	CHINA-YAAS-2010-106	China	Breeding line
5	IT 100414	*Brassica rapa* L.	Turnip	AVRDC-KJH-1985-100414	Taiwan	-
6	IT 260820	*Brassica rapa* L.	Kimchi cabbage	CHINA-YAAS-2010-107	China	Breeding line
7	IT 100416	*Brassica rapa* L.	Kimchi cabbage	AVRDC-KJH-1985-100416	Taiwan	-
8	IT 100413	*Brassica rapa* L.	Turnip	AVRDC-KJH-1985-100413	Taiwan	-
9	IT 100388	*Brassica rapa* L.	Kimchi cabbage	AVRDC-KJH-1985-100388	Taiwan	-
10	IT 100408	*Brassica rapa* L.	Kimchi cabbage	AVRDC-KJH-1985-100408	Taiwan	-
11	IT 260824	*Brassica rapa* L.	Kimchi cabbage	CHINA-YAAS-2010-111	China	Breeding line
12	IT 228167	*Brassica rapa* L.	Kimchi cabbage	36197	Taiwan	-
13	IT 100404	*Brassica rapa* L.	Kimchi cabbage	AVRDC-KJH-1985-100404	Taiwan	-
14	IT 100352	*Brassica rapa* L.	Kimchi cabbage	AVRDC-KJH-1985-100352	Taiwan	-
15	IT 293231	*Brassica rapa* L.	Kimchi cabbage	WIR68	Ethiopia	Cultivar
16	IT 100412	*Brassica rapa* L.	Kimchi cabbage	AVRDC-KJH-1985-100412	Taiwan	-
17	IT 100411	*Brassica rapa* L.	Kimchi cabbage	AVRDC-KJH-1985-100411	Taiwan	-
18	IT 100371	*Brassica rapa* L.	Kimchi cabbage	AVRDC-KJH-1985-100371	Taiwan	-
19	IT 135409	*Brassica rapa* L.	Kimchi cabbage	Shingatsuna	Japan	Landrace
20	IT 32750	*Brassica rapa* L.	Kimchi cabbage	Ching Pao 26	China	Cultivar
21	IT 100406	*Brassica rapa* L.	Mibuna	AVRDC-KJH-1985-100406	Taiwan	
22	IT 100353	*Brassica rapa* L.	Kimchi cabbage	AVRDC-KJH-1985-100353	Taiwan	
23	IT 100372	*Brassica rapa* L.	Kimchi cabbage	AVRDC-KJH-1985-100372	Taiwan	
24	Commercial	*Brassica rapa* L.	Kimchi cabbage	Hangamssam 1	South Korea	Cultivar
25	IT 100366	*Brassica rapa* L.	Kimchi cabbage	AVRDC-KJH-1985-100366	Taiwan	
26	IT 100393	*Brassica rapa* L.	Leaf mustard	AVRDC-KJH-1985-100393	Taiwan	
27	IT 100395	*Brassica rapa* L.	Kimchi cabbage	AVRDC-KJH-1985-100395	Taiwan	
28	IT 163625	*Brassica rapa* L.	Kimchi cabbage	Yeongdeog Sandongchae-2	South Korea	Landrace
29	Commercial	*Brassica rapa* L.	Kimchi cabbage	Weoldongdaewang	South Korea	Cultivar
30	IT 199678	*Brassica rapa* L.	Kimchi cabbage	WIR33507	China	Landrace
31	IT 199706	*Brassica rapa* L.	Kimchi cabbage	WIR30643	China	Landrace
32	IT 32733	*Brassica rapa* L.	Kimchi cabbage	Song Dao Xin 2	China	Cultivar
33	IT 32738	*Brassica rapa* L.	Kimchi cabbage	Weonsi-1984-Kimchicabbage32738	South Korea	-
34	IT 219574	*Brassica rapa* L.	Kimchi cabbage	Kang re jieqiuxiayangbaoxinbai 50 tian	China	Cultivar
35	IT 262102	*Brassica rapa* L.	Kimchi cabbage	Namyeon1-ho	North korea	Cultivar
36	IT 100383	*Brassica rapa* L.	Kimchi cabbage	AVRDC-KJH-1985-100383	Taiwan	-
37	IT 120112	*Brassica rapa* L.	Kimchi cabbage	Shuang Ching 156	China	Cultivar
38	IT 163707	*Brassica rapa* L.	Kimchi cabbage	JangsuSandongchae	South Korea	Landrace
39	IT 166984	*Brassica rapa* L.	Kimchi cabbage	Tianjin qing	China	Landrace
40	IT 163708	*Brassica rapa* L.	Kimchi cabbage	Muju Sandongchae1	South Korea	Landrace
41	Commercial	*Brassica rapa* L.	Kimchi cabbage	Balgang 3-ho	South Korea	Cultivar
42	Commercial	*Brassica rapa* L.	Kimchi cabbage	Alchandul	South Korea	Cultivar
43	IT 215003	*Brassica rapa* L.	Kimchi cabbage	Jeonnam Haenam-2000-36	South Korea	Landrace
44	IT 199670	*Brassica rapa* L.	Kimchi cabbage	Dak-se	South Korea	Landrace
45	IT 206799	*Brassica oleracea* L.	Cabbage	NPL-KIG-1997-278	South Korea	-
46	IT 216342	*Brassica rapa* L.	Kimchi cabbage	Baoshou 3	China	Cultivar
47	IT 100409	*Brassica juncea* L. Czern.	Leaf mustard	AVRDC-KJH-1985-100409	Taiwan	-
48	Commercial	*Brassica rapa* L.	Kimchi cabbage	Hangamssam2	South Korea	Cultivar

* Scientific names of each plant are assigned based on the status given on http://www.theplantlist.org. Only accepted names are used.

**Table 2:** List of identified glucosinolates, retention time (RT), calibration curves, and multiple reaction monitoring (MRM) conditions for quantitation of glucosinolates by negative ion MRM (see Supplementary File (Figure S1) for chromatogram).

Glucosinolates	RT (min)	MRM Transition	CID (ev)	Dwell Time (sec)	Calibration Curve Parameters
Progoitrin (PRO)	1.41	387.77 > 194.85	20	0.033	Y = 3.59902X – 20.5808 (r^2^ = 0.999)
Gluconapin (GNA)	3.02	371.74 > 258.74	20	0.033	Y = 3.50074X + 3.51886 (r^2 =^ 0.996)
Glucobrassicanapin (GBN)	4.42	385.71 > 258.87	25	0.033	Y = 2.68899X – 2.8434 (r^2^ = 0.994)
Glucotropaeolin (TRO)	4.84	407.72 > 258.87	20	0.033	Y = 6.27084X – 4.49552 (r^2^ = 0.999)
Glucoerucin (ERU)	4.97	419.69 > 258.74	25	0.033	Y = 2.41077X + 16.6315 (r^2^ = 0.999)
Glucobrassicin (GBC)	5.61	446.69 > 204.94	20	0.033	Y = 1.76969X – 11.3033 (r^2^ = 0.999)
Glucoberteroin (BER)	6.29	433.72 > 275.06	20	0.033	Y = 2.92616X – 3.54071 (r^2^ = 0.993)
Gluconasturtiin (NAS)	6.33	421.69 > 274.87	25	0.033	Y = 1.98894X + 1.81048 (r^2^ = 0.994)

CID = collision-induced dissociation; LOQ = limit of quantification; Pol. = polarity.

**Table 3:** Glucosinolate content (µmol kg^−1^ DW) in 48 germplasm accessions of *Brassica* (n = 3).

S/No	Gluconapin	Glucobrassicanapin	Progoitrin	Glucotropaeolin	Glucoerucin	Gluconasturtiin	Glucoberteroin	Glucobrassicin	Sum
1	59.88 ± 9.48A	14,961.04 ± 64R	1191.24 ± 46.86U	21.08 ± 0.36P	59.85 ± 4.55L	3713.98 ± 118.91Q	450.56 ± 8.05P	1257.38 ± 46.19M	21,715.00 ± 186.56Q
2	13,634.53 ± 32.45S	19,279.35 ± 711.33V	1548.95 ± 12.76X	20.96 ± 1.63OP	39.21 ± 2.26J	6307.25 ± 365.08W	664.07 ± 34.97R	939.88 ± 77.28KL	42,434.21 ± 1042.22X
3	24.07 ± 2.22A	19,737.72 ± 527.7W	1207.7 ± 23.17UV	18.96 ± 1.38OP	20.73 ± 0.51H	4701.79 ± 91.65T	247.37 ± 7.70K-M	2352.8 ± 65.59Q	28,311.15 ± 639.34V
4	54.11 ± 7.74A	18,361.17 ± 307.39U	1979.79 ± 43.03Y	9.13 ± 0.46JK	116.8 ± 2.76Q	6353.11 ± 137.87W	1221.33 ± 13.01T	909.34 ± 15.88KL	29,004.78 ± 212.28V
5	11.9 ± 0.61A	5877.32 ± 85.72HI	13.78 ± 0.19AB	31.77 ± 1.12R	10.3 ± 0.62E-G	1070.32 ± 20.69HIJ	17.19 ± 1.18AB	375.17 ± 24.16C-E	7407.74 ± 124.18FG
6	3476.23 ± 182.91lM	13,029.46 ± 135.44P	147.35 ± 5.72I	6.21 ± 0.71F-I	27.7 ± 2.97I	5471.57 ± 147.53V	310.33 ± 5.7NO	633.73 ± 27.11G-I	23,102.58 ± 347ST
7	4735.79 ± 147.57P	17,678.49 ± 118.58T	30.92 ± 2.36ABC	8.14 ± 1.13IJ	3.48 ± 0.31A-E	1808.78 ± 49.56L	52.03 ± 2.5A-F	967.2 ± 9.61KL	25,284.81 ± 204.38U
8	15,276.5 ± 3.34T	6141.34 ± 63.04HI	109.45 ± 6.7G	8.91 ± 0.17JK	3.55 ± 0.22A-E	635.54 ± 15.93DEF	16.21 ± 0.23AB	413.84 ± 4.04D-F	22,605.35 ± 44.95RS
9	3728.9 ± 164.19M	13,101.21 ± 394.68P	560.33 ± 11.65O	15.61 ± 1.58N	4.74 ± 0.23A-E	3677.9 ± 184.07Q	61.98 ± 1.17B-G	1623.02 ± 116.35N	22,773.70 ± 830.86R-T
10	2946.79 ± 232.69K	9778.47 ± 286.18M	32.95 ± 1.13A-D	21.01 ± 0.89OP	2.76 ± 0.2A-D	918.89 ± 33.45GHI	13.13 ± 1.09AB	278.44 ± 8.22A-D	13,992.44 ± 545.46L
11	3299.16 ± 59L	9673.08 ± 198.58LM	296.33 ± 10.91L	2.3 ± 0.1A-C	7.61 ± 0.17B-F	4023.25 ± 75.16R	160.08 ± 3.25I	821.22 ± 19.58I-K	18,283.02 ± 281.06O
12	3535.26 ± 199.95lM	5062.22 ± 88.73GH	12.64 ± 1.72AB	7.87 ± 0.42H-J	725.93 ± 10.93U	3134.41 ± 121.18O	2574.83 ± 88.13Y	842.07 ± 20.2I-K	15,895.23 ± 287.36M
13	4191.84 ± 46.19NO	9971.01 ± 269.09M	331.44 ± 19.07L	14.61 ± 1.27MN	3.25 ± 0.35A-E	643.2 ± 12.04DEF	78.21 ± 1.74C-H	500.03 ± 11.73E-G	15,733.6 ± 283.92M
14	3336.34 ± 165.05L	13,541.59 ± 96.59Q	474.05 ± 17.5N	9.05 ± 1.17JK	5.92 ± 0.2A-E	2722.06 ± 114.26N	85.32 ± 1.8D-H	547.25 ± 7.77E-G	20,721.58 ± 158.21P
15	3467.66 ± 112.06LM	6626.38 ± 172.30J	227.4 ± 4.68K	4.03 ± 0.51B-F	76.69 ± 0.99MN	4261.8 ± 210.19S	279.83 ± 7.15L-N	603.83 ± 27.82F-H	15,547.64 ± 447.31M
16	4010.97 ± 74.12N	3497.18 ± 12.57EF	165.63 ± 1.76I	8.18 ± 0.28IJ	85.21 ± 2.39O	1773.78 ± 68.97L	166.24 ± 2.24IJ	1088.08 ± 34.99LM	10,795.26 ± 175.08J
17	11.93 ± 2.53A	2924.5 ± 44.36DE	111.5 ± 4.38GH	29.87 ± 1.33R	85.48 ± 6.99O	858.03 ± 28.63F-H	236.7 ± 11.87KL	546.02 ± 33.92E-G	4804.03 ± 115.95D
18	2070.69 ± 49J	9270.47 ± 118.15L	918.9 ± 12.68T	20.1 ± 0.32OP	125.37 ± 0.74R	4700.27 ± 183.2T	1616.48 ± 36.22V	3206.66 ± 111.92S	21,928.94 ± 399.73QR
19	21.75 ± 4.52A	15,704.45 ± 392.29S	417.99 ± 1.94M	11.73 ± 0.51L	204.85 ± 6.86T	4700.35 ± 145.32T	457.36 ± 8.04P	1953.32 ± 63.03O	23,471.8 ± 375.26T
20	1190.43 ± 38.54FG	6263.87 ± 114.31HIJ	1474.34 ± 9.07W	39.75 ± 0.45S	106.73 ± 2.46P	3441.64 ± 180.17P	806.95 ± 12.97S	12134.4 ± 474.88U	25,458.11 ± 810.62U
21	2973.98 ± 57.69k	10,636.42 ± 86.88N	96.36 ± 4.52FG	18.51 ± 0.65O	3.26 ± 0.41A-E	2264.32 ± 39.6M	49.01 ± 2.14A-F	647.84 ± 17.57G-J	16,689.69 ± 163.39N
22	5144.32 ± 213.54q	1101.59 ± 71.18B	0.52 ± 0.05A	12.04 ± 0.66L	0.76 ± 0.06AB	46.6 ± 5.15A	0.19 ± 0.01A	186.51 ± 12A-C	6492.51 ± 297.72E
23	1602.12 ± 31.52hi	10,590.99 ± 161.77N	873.97 ± 13.86S	29.87 ± 0.55R	78.11 ± 5.31N	5047.2 ± 100.45U	1494.98 ± 35.08U	2955.72 ± 34.85R	22,672.94 ± 351.75R-T
24	2866.29 ± 596.67K	9938.31 ± 179.31M	626.3 ± 13.28P	15.83 ± 0.68N	188.01 ± 6.86S	2814.85 ± 71.76N	2426.77 ± 72.17W	1256.82 ± 29.37M	20,133.19 ± 869.75P
25	1266.69 ± 23.11FG	11,840.19 ± 410.97O	795.24 ± 21.51R	57.1 ± 3.27U	48.10 ± 1.20K	5079.75 ± 283.32U	663.97 ± 26.40R	2310.74 ± 95.76PQ	22,061.78 ± 819.36QR
26	10,185.75 ± 257.48R	23,026.64 ± 620.66X	240.42 ± 10.62K	5.41 ± 0.57D-H	0.47 ± 0.03AB	3414.3 ± 90.05P	70.51 ± 2.29B-H	510.19 ± 9.12E-G	37,453.7 ± 927.2W
27	4356.59 ± 95.73O	2446.97 ± 29.44C	15.49 ± 1.39AB	1.08 ± 0.17A	9.21 ± 1.25C-H	306.57 ± 10.02BC	21.45 ± 0.6A-C	174.01 ± 3.42A-C	7331.37 ± 125.6EF
28	1395.66 ± 27.59GH	8532.77 ± 182.94K	941.11 ± 43.58T	13.42 ± 0.41lMN	15.45 ± 2.45GH	1008.44 ± 20.59HIJ	215.08 ± 8.93JK	369.49 ± 21.95C-E	12,491.42 ± 280.55K
29	489.47 ± 9.08DE	6278.88 ± 54.44H-J	1240.81 ± 21.58V	11.2 ± 0.81KL	5.24 ± 0.65A-E	2180.63 ± 94.1M	112.39 ± 0.96G-I	3089.58 ± 115.3RS	13,408.2 ± 184.56L
30	255.83 ± 11.73A-D	3853.9 ± 24.7F	619.18 ± 23.47P	10.9 ± 1.15KL	71.37 ± 1.69M	1179.72 ± 17.94J	802.45 ± 24.23S	2944.82 ± 114.14R	9738.15 ± 97.86I
31	257.55 ± 8.54A-D	2780.93 ± 77.85CD	561.96 ± 10.64O	47.77 ± 5.06T	56.59 ± 2.96L	1514.37 ± 95.45K	658.95 ± 42.59R	2141.2 ± 140.05OP	8019.32 ± 348.92F-H
32	544.53 ± 6.47E	3671.15 ± 20.04F	170.87 ± 12.02I	13.03 ± 0.67LM	0.29 ± 0.00A	858.07 ± 43.68F-H	17.74 ± 1.92AB	1995 ± 80.33O	7270.68 ± 126.13EF
33	1074.06 ± 29.14F	4647.27 ± 136.25GH	207.8 ± 40.27JK	6.08 ± 0.44E-I	2.44 ± 0.55A-C	1466.53 ± 33.59K	44.76 ± 0.61A-F	774.18 ± 36.47H-K	8223.13 ± 263.56GH
34	466.79 ± 1.68C-E	3113.24 ± 68.28DE	169.68 ± 7.9I	7.85 ± 0.36HIJ	15.06 ± 2.73GH	1026.78 ± 23.48H-J	111.86 ± 4.39G-I	2335.53 ± 123.13PQ	7246.78 ± 214.09EF
35	51.05 ± 1.94A	305.54 ± 12.68A	39.96 ± 6.33BCD	7.73 ± 0.53HIJ	2.13 ± 0.27A-C	891.29 ± 31.8GH	36.19 ± 0.89A-E	3054.82 ± 161.62RS	4388.7 ± 197.89C
36	194.61 ± 8.14A-C	1402.18 ± 28.1B	95.62 ± 7.09FG	4.68 ± 0.45C-G	9.22 ± 1.13C-G	477.73 ± 14.12C-E	87.78 ± 1.76D-H	1759.17 ± 53.61N	4030.99 ± 61.51CD
37	126.23 ± 1.92AB	1419.5 ± 21.17B	177.16 ± 3.65IJ	6.94 ± 0.99GHIJ	3.51 ± 0.39A-E	697.43 ± 29.58E-G	94.04 ± 2.96E-H	1280.23 ± 46.13M	3805.05 ± 83.11C
38	592.17 ± 12.37E	6582.48 ± 116.76IJ	753.42 ± 15.53Q	3.59 ± 0.46A-E	14.2 ± 1.19FG	1241.58 ± 60.26J	352.78 ± 3.66O	263.48 ± 10.36A-D	9803.7 ± 116.59I
39	381.38 ± 9.82B-E	2785.14 ± 49.21CD	300.07 ± 6.27L	3.06 ± 0.25A-D	77.62 ± 3.87N	1136.92 ± 10.75IJ	526.19 ± 20.32Q	3186.07 ± 86.65S	8396.45 ± 101.55H
40	1791.19 ± 29.09I	11,617.64 ± 110.72O	391.31 ± 23.16M	4.02 ± 0.33B-F	3.01 ± 0.22A-D	3232.81 ± 41.13OP	103.98 ± 5.05F-H	944.95 ± 13.14KL	18,088.92 ± 172.28O
41	70.72 ± 3.11A	1309.85 ± 21.19B	144.92 ± 14.72HI	2.47 ± 0.49A-C	5.19 ± 0.53A-E	443.64 ± 11.57CD	294.7 ± 7.18MN	139.05 ± 6.11AB	2410.54 ± 48.13B
42	15.71 ± 1.75A	210.42 ± 2.84A	69.41 ± 14.23D-F	6.01 ± 0.29E-I	6.5 ± 0.99A-E	161.18 ± 2.06AB	31.09 ± 1.34A-D	120.81 ± 7.66A	621.15 ± 10.07A
43	33.68 ± 2.34A	216.5 ± 2.46A	58.56 ± 5.03C-E	1.97 ± 0.17AB	9.97 ± 0.79D-G	145.54 ± 6.6AB	123.9 ± 6.94HI	207.89 ± 11.57A-D	798.00 ± 14.86A
44	27.63 ± 4.55A	282.4 ± 5.17A	26.03 ± 3.18ABC	1.04 ± 0.26A	4.36 ± 0.42A-E	181.09 ± 5.68AB	67.08 ± 3.94B-H	326.18 ± 20.08A-E	915.81 ± 20.17A
45	105.54 ± 2.09AB	0.04 ± 0.01A	42.05 ± 0.57B-D	1.16 ± 0.27A	ND	302.73 ± 6.55BC	0.04 ± 0.01A	856.47 ± 49.15J-L	1308.02 ± 43.14A
46	21.56 ± 0.84A	67.31 ± 1.05A	88.33 ± 8.34E-G	8.13 ± 0.39IJ	1.53 ± 0.21AB	291.44 ± 12.69BC	20.23 ± 2.05A-C	350.9 ± 25.66B-E	849.43 ± 30.04A
47	35.79 ± 1.8A	1.68 ± 0.06A	0.32 ± 0.08A	11.25 ± 0.48KL	ND	681.72 ± 11.04E-G	0.05 ± 0.01A	121.37 ± 8.89A	852.18 ± 21.09A
48	233.33 ± 5.72A-D	946.67 ± 19.13B	801.03 ± 16.99R	24.5 ± 0.91Q	ND	1610.44 ± 50.06KL	2510.9 ± 94.18X	3547.13 ± 191.53T	9673.98 ± 326.32I

Values are mean ± standard deviation of biological triplicates. Different letters between rows indicate statistically significant differences at *p* < 0.05. S/No corresponds to the genetic resources described in Table 1.

## Supplementary Materials

The following are available online at https://www.mdpi.com/2223-7747/9/11/1421/s1, Figure S1: A representative MRM profile of the mixture of glucosinolates. Figure S2: Principal component analysis (PCA) plot of the scores generated based on the country of origin for 45 samples and nine glucosinolates (Chinese origin, 13 accessions; South Korean origin, 12 accessions; and Taiwanese origin, 20 accessions). Table S1: Glucosinolate concentration in different leaf sections of three cultivars of kimchi cabbage (µmol kg^−1^ DW). Table S2: Loadings, eigenvalues, and percentage of variance for the principal components (PCs) data from germplasm collections.
