# Supplementary material for: Glucosinolate Content in Brassica Genetic Resources and Their Distribution Pattern within and between Inner, Middle, and Outer Leaves
Source: Plants (Basel). 2020 Oct 23;9(11):1421. doi: 10.3390/plants9111421 (PMC7690824; doi:10.3390/plants9111421)
Supplement: Supplementary file 1 [file plants-09-01421-s001.pdf]

Glucosinolate content in *Brassica* Genetic Resources and Their Distribution Pattern within and between Inner, Middle, and Outer Leaves

Ju-Hee Rhee[1], Susanna Choi[1], Jae-Eun Lee[1], On-Sook Hur[1], Na-Young Ro[1], Ae-Jin Hwang[1], Ho-Cheol Ko[2], Yun-Jo Chung[3], Jae-Jong Noh[4], Awraris Derbie Assefa[1]*

[1] National Agrobiodiversity Center, National Institute of Agricultural Sciences, RDA, Jeonju 54874, Rep. of Korea; rheehk@korea.kr (J.H.R.); protect46284628@gmail.com (S.C.); jnlee88@korea.kr (J.E.L.); oshur09@korea.kr (O.S.H.); nonanona@korea.kr (N.Y.R.); hyj6138@korea.kr (A.J.H.)

[2] Client Service Division, Planning and Coordination Bureau, RDA, Jeonju 54875, Rep. of Korea; hchko@korea.kr

[3] National Creative Research Laboratory for Ca^{2+} signaling Network, Jeonbuk National University Medical School, Jeonju, 54896, Rep. of Korea; yjchong@jbnu.ac.kr

[4] Jeonbuk Agricultural Research and Extension Services, Iksan 54591, Korea; nohjj@korea.kr

* Corresponding author: awraris@korea.kr; Tel.: +82-63-238-4902

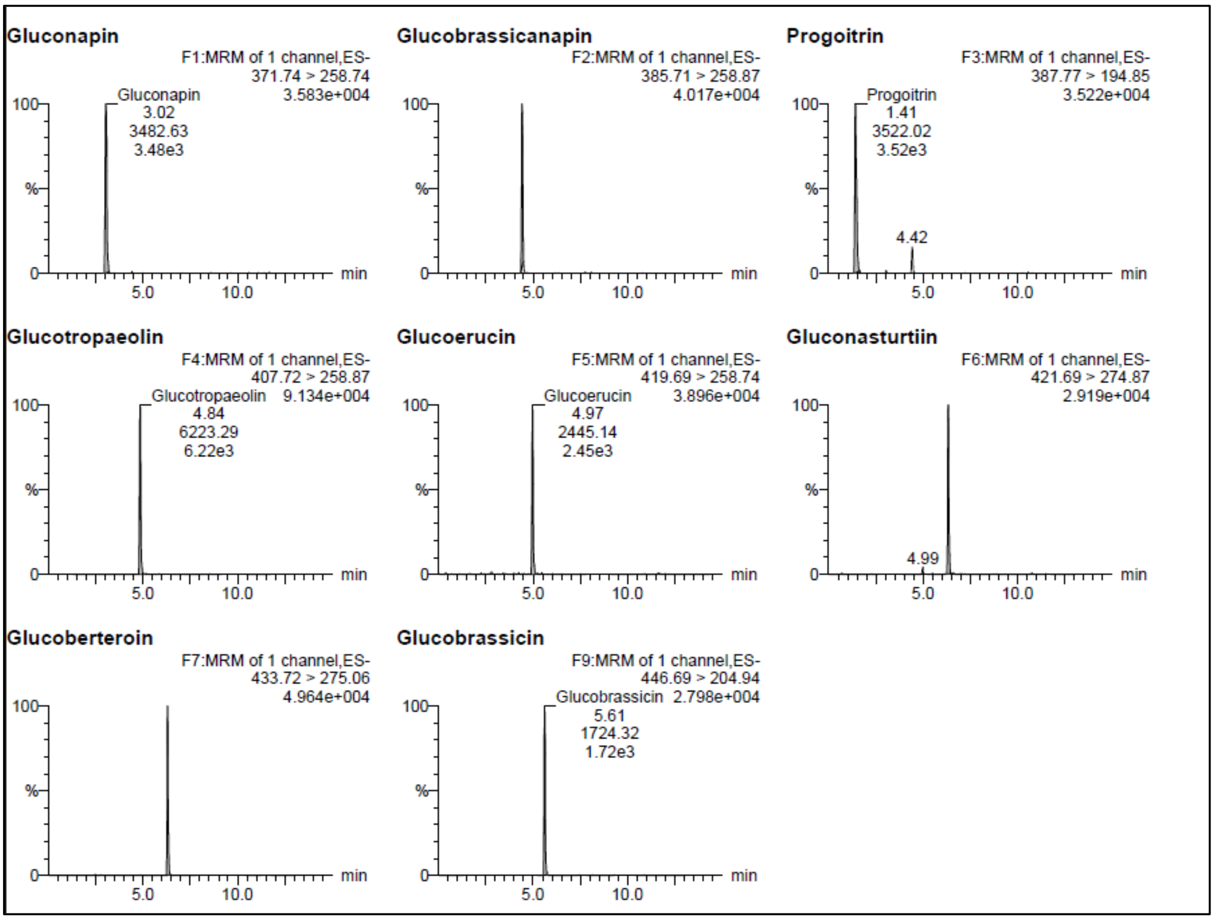

Figure S1. A representative MRM profiles of a mixture of glucosinolates

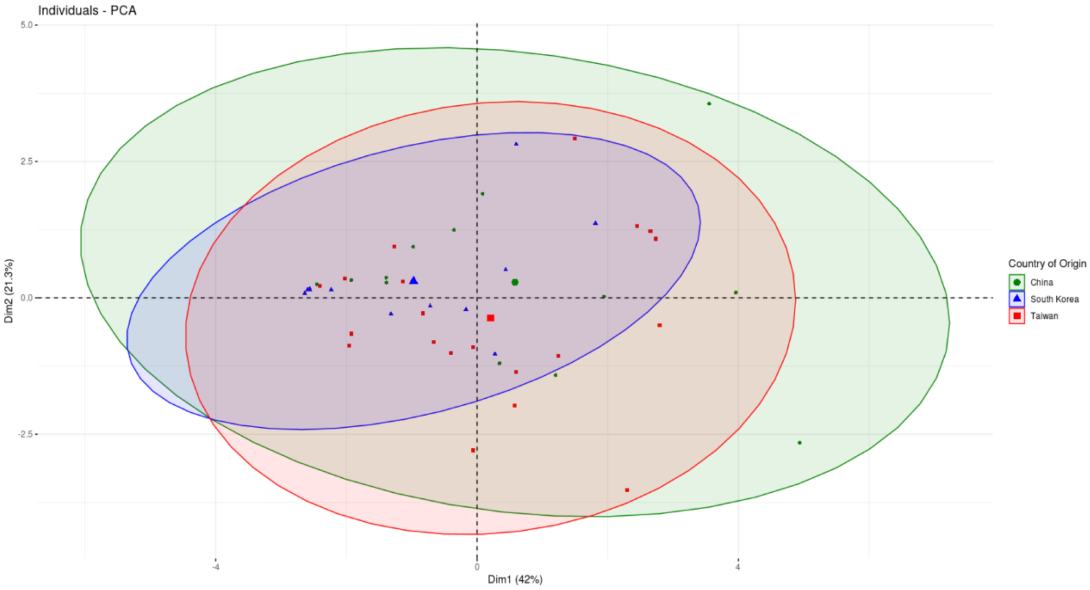

Figure S2. Principal Component Analysis (PCA) plot of the scores generated based on the country of origin 45 samples and eight glucosinolates (Chinese origin 13 accessions; South Korean origin 12 accessions; and Taiwanese origin 20 accessions).

Table S1. Glucosinolate concentration in different leaf sections of three cultivars of kimchi cabbage (µmol kg^{-1} DW)

Cultivar, leaf location, and part			Gluconapin	Glucobrassicanapin	Progoitrin	Glucotropaeolin	Glucoerucin	Gluconasturtiin	Glucoberteroin	Glucobrassicin	Sum
'Hangamssam'	Outer layer	top	961.07 ± 17.59A	2976.34 ± 273.91A	170.92 ± 15.37A	3.44 ± 0.69C	ND	425.44 ± 35.81A	6.80 ± 0.90A	98.82 ± 14.51A	4642.82 ± 346.16A
		middle	3259.58 ± 91.12F	8979.31 ± 180.02F	529.83 ± 17.00D	5.08 ± 0.4D	35.10 ± 2.17B	1483.69 ± 4.69DE	1134.90 ± 18.78E	141.59 ± 2.43A	15,569.08 ± 100.01F
		bottom	2092.19 ± 35.75BC	7586.82 ± 166.22E	727.56 ± 17.82F	2.00 ± 0.23B	62.92 ± 2.89C	1638.75 ± 87.38E	953.66 ± 42.15D	240.41 ± 2.98B	13,304.31 ± 108.23D
		green	1224.95 ± 29.7A	4057.55 ± 132.11B	429.17 ± 22.98C	3.06 ± 0.81C	ND	503.20 ± 26.58A	9.37 ± 0.07A	115.09 ± 9.85A	6342.37 ± 211.14B
		white	1801.29 ± 53.86B	9518.23 ± 123.66FG	1347.05 ± 23.43I	0.75 ± 0.13A	34.45 ± 0.59B	772.50 ± 36.50B	747.30 ± 30.18C	148.33 ± 8.12A	14,369.90 ± 182.25E
	Middle layer	top	2808.58 ± 233.54E	5284.53 ± 325.21C	331.13 ± 38.44B	4.67 ± 0.15D	0.60 ± 0.02A	1401.43 ± 139.04D	82.41 ± 8.72AB	314.54 ± 4.58C	10,227.89 ± 698.69C
		middle	3630.65 ± 300.37G	9301.03 ± 631.85FG	629.81 ± 23.8E	11.53 ± 0.35F	55.61 ± 3.00C	2167.80 ± 161.71F	1362.39 ± 95.07F	485.42 ± 36.19E	17,644.23 ± 1187.92GH
		bottom	2602.54 ± 108.58DE	9700.8 ± 186.89G	973.52 ± 21.04H	2.01 ± 0.40B	122.93 ± 6.38D	1627.81 ± 85.39E	1184.52 ± 37.30E	676.75 ± 46.22F	16,890.88 ± 384.78G
		green	2372.27 ± 89.53CD	6073.86 ± 113.96D	585.01 ± 20.8E	7.22 ± 0.18E	0.78 ± 0.14A	1185.22 ± 41.34C	117.30 ± 3.69B	295.90 ± 21.00C	10,637.58 ± 178.74C
		white	3216.77 ± 220.12F	9594.51 ± 87.52G	876.46 ± 28.6G	1.29 ± 0.34AB	186.48 ± 8.97E	2210.61 ± 60.81F	1766.32 ± 53.87G	405.06 ± 26.06D	18,257.50 ± 306.12H
'Bbalgang 3-ho'	Outer layer	top	3.01 ± 0.23A	105.85 ± 5.79A	46.97 ± 5.37A	8.55 ± 0.41F	ND	23.26 ± 1.39A	0.22 ± 0.05A	92.68 ± 4.83B	280.54 ± 4.62A
		middle	49.35 ± 2.73A	1128.13 ± 47.65D	127.36 ± 4.78C	3.23 ± 0.33C	0.87 ± 0.01A	249.02 ± 11.27C	54.69 ± 2.62A	277.42 ± 13.88F	1890.07 ± 75.39C
		bottom	145.59 ± 2.97C	1490.53 ± 25.55F	230.74 ± 8.82E	4.44 ± 0.14D	17.02 ± 3.15B	364.55 ± 16.06D	696.68 ± 23.81D	81.06 ± 2.3B	3030.62 ± 11.57D
		red	292.82 ± 7.86D	3540.52 ± 41.99J	625.34 ± 19.51H	6.06 ± 1.12E	18.22 ± 1.64B	645.26 ± 24.94G	1325.40 ± 59.85F	117.26 ± 3.8C	6570.88 ± 140.64H
		white	123.27 ± 18.68BC	1224.06 ± 12.96E	96.41 ± 15.76BC	0.66 ± 0.08A	2.23 ± 0.46A	271.94 ± 15.21C	232.08 ± 7.94B	32.35 ± 1.47A	1983.01 ± 33.15C
	Middle layer	top	506.77 ± 40.38E	4872.67 ± 117.57K	659.7 ± 27.47I	2.81 ± 0.22C	154.16 ± 14.64D	975.28 ± 26.48H	3840.76 ± 49.33H	349.85 ± 17.81H	11,362 ± 143.39J
		middle	65.51 ± 2.21AB	509.82 ± 15.11C	92.58 ± 15.95B	0.13 ± 0.02A	7.33 ± 0.33A	75.63 ± 2.57B	357.55 ± 8.33C	20.89 ± 2.23A	1129.43 ± 3.14B
		bottom	252.55 ± 55.59d	2275.00 ± 54.01H	239.11 ± 6.79E	1.00 ± 0.25AB	18.32 ± 1.66B	546.08 ± 10.98F	707.86 ± 12.24D	144.10 ± 8.45E	4184.01 ± 31.5F
		red	267.45 ± 38.39D	3225.86 ± 37.61I	537.76±34.43G	21.63 ± 0.7G	10.21 ± 0.42AB	581.79 ± 11.89F	972.40 ± 10.77E	123.67 ± 8.95CD	5740.76 ± 61.38G
		white	572.76 ± 58.79F	4803.81 ± 27.14K	428.69 ± 1.9F	5.10 ± 0.56D	33.88 ± 0.98C	1097.35 ± 47.77I	1690.15 ± 62.53G	306.17 ± 13.09G	8937.93 ± 36.55I
	Inner layer	top	14.29 ± 3.14A	197.10 ± 9.75B	21.68 ± 2.23A	0.15 ± 0.01A	0.78 ± 0.05A	43.15 ± 2.03AB	40.97 ± 1.14A	37.68 ± 3.72A	355.81 ± 15.19A
		middle	170.39 ± 16.69C	1995.48 ± 23.93G	178.2 ± 9.41D	1.58 ± 0.06B	18.23 ± 1.31B	420.53 ± 34.63E	677.72 ± 33.46D	405.74 ± 18.78I	3867.88 ± 104.54E
		bottom	74.74 ± 12.81AB	1081.97 ± 12.92D	103.01 ± 6.79BC	1.72 ± 0.25B	4.26 ± 0.79A	271.24 ± 10.74C	200.00 ± 9.42B	142.57 ± 2.83DE	1879.52 ± 23.76C
		red	8.63 ± 1.83A	227.44 ± 6.54B	25.07 ± 1.67A	0.09 ± 0.01A	ND	46.14 ± 0.29AB	28.61 ± 0.55A	76.16 ± 3.96B	412.13 ± 13.04A

		white	619.9 ± 64.98F	5791.84 ± 28.69L	755.87 ± 14.31J	3.07 ± 0.48C	167.2 ± 3.24E	1231.21 ± 33.87J	4478.94 ± 135.63I	579.80 ± 7.70J	13,627.82 ± 250.95K
'Alchandul'	Outer layer	top	109.6 ±2 4.79CD	1217.35 ± 32.22C	870.94 ± 7.41F	8.55 ± 0.46C	4.22 ± 0.59A	617.28 ± 25.05B	207.97 ± 16.49B	164.14 ± 6.13A-C	3200.06 ± 67.02B
		middle	221.83 ± 13.84E	2049.31 ± 64.41F	980.3 ± 14.82G	9.99 ± 0.68C-E	183.54 ± 6.63F	1005.20 ± 62.49D	1439.94 ± 78.48E	160.22 ± 5.03A-C	6050.33 ± 190.78F
		bottom	59.19 ± 2.39AB	1362.08 ± 24.02D	588.02 ± 8.79D	36.63 ± 1.04H	48.46 ± 4.88C	1475.97 ± 42.87G	147.21 ± 4.45AB	4537.91 ± 253.09H	8255.47 ± 301.84I
		green	16.77 ± 0.75A	259.58 ± 5.66A	507.66 ± 6.06C	6.15 ± 0.32B	5.38 ± 0.05A	232.01 ± 9.95A	133.56 ± 9.27A	48.41 ± 0.47A	1209.52 ± 20.07A
		white	370.31 ± 44.98G	2950.92 ± 49.75I	1253.7 ± 2.62J	5.31 ± 0.45AB	29.71 ± 1.16B	1374.76 ± 10.96F	458.68 ± 9.35D	333.41 ± 11.04D	6776.8 ± 102.59G
	Middle layer	top	121.08 ± 7.31CD	1186.09 ± 17.94C	1304 ± 26.5K	6.88 ± 0.31B	611.55 ± 9.59K	1177.56 ± 12.01E	2818.83 ± 41.36H	273.6 ± 4.54B-D	7499.59 ± 100.88H
		middle	383.05 ± 39.62G	3049.15 ± 7.37J	1534.15 ± 13.5M	10.74 ± 0.23E	589.76 ± 5.31J	1652.92 ± 7.71H	3087.86 ± 41.84I	354.4 ± 9.07D	10662.03 ± 82.38J
		bottom	215.07 ± 43.18E	2155.94 ± 52.88G	1213.13 ± 24.72I	10.69 ± 1.12E	195.58 ± 9.25G	1335.94 ± 45.03F	1437.95 ± 32.17E	333.35 ± 13.7D	6897.63 ± 194.96G
		green	76.85 ± 6.2BC	805.66 ± 16.6B	1037.17 ± 5.85H	3.88 ± 0.44A	420.92 ± 1.89I	1019.83 ± 15.49D	1889.66 ± 45.15G	125.86 ± 5.08AB	5379.84 ± 50.23D
		white	282.45 ± 18.35F	2503.95 ± 36.03H	1426.77 ± 30.93L	10.51 ± 0.43DE	231.96 ± 7.51H	1674.70 ± 15.8H	1753.66 ± 22.54F	311.51 ± 6.80CD	8195.52 ± 79.31I
	Inner layer	top	124.08 ± 12.03CD	1385.71 ± 25.82D	355.67 ± 10.5A	8.90 ± 0.21CD	107.32 ± 1.98E	897.58 ± 26.46C	451.6 ± 12.38D	1549.72 ± 38.59F	4880.6 ± 49.26C
		middle	177.42 ± 7.28E	1810.68 ± 51.39E	474.76 ± 4.48B	16.22 ± 1.49F	77.88 ± 4.69D	1156.49 ± 47.33E	353.37 ± 7.22C	1702.39 ± 29.06G	5769.2 ± 101.01E
		bottom	129.72 ± 1.89D	1979.55 ± 13.5F	785.1 ± 5.62E	29.76 ± 1.32G	98.53 ± 2.63E	1330.49 ± 51.63F	477.79 ± 5.23D	1145.55 ± 40.00E	5976.49 ± 61.46EF

Values are Mean ± standard deviation of biological triplicates. Different letters between rows within each sample indicate statistically significant differences at $p < 0.0$

Table S2. Loadings, eigenvalues, and percentage of variance for the principal components (PCs) data from germplasm collections

Compound	PC 1	PC 2	PC 3	PC 4	PC 5	PC 6	PC 7	PC 8
GNA	0.086336	0.54354	0.2008	0.70637	0.328	-0.17544	0.069747	-0.12083
GBN	0.3783	0.51247	-0.03552	-0.17494	-0.14352	0.26339	-0.06507	0.68425
PRO	0.46858	0.016063	-0.27461	-0.28142	0.21388	-0.48929	0.57936	-0.069
TRO	0.31799	-0.22723	-0.3494	0.53154	-0.65751	0.018292	0.097071	-0.02358
ERU	0.2445	-0.25094	0.67441	0.037057	-0.03716	0.408	0.50281	-0.01979
NAS	0.49583	0.24908	0.029853	-0.24234	-0.08117	0.23516	-0.36189	-0.66305
BER	0.37089	-0.33185	0.42749	0.042825	0.00672	-0.52589	-0.48607	0.23424
GBC	0.29251	-0.39359	-0.34995	0.21624	0.62108	0.40222	-0.16057	0.13035
Eigenvalues	2.99747	1.67073	1.31784	0.710531	0.571138	0.383894	0.199721	0.148675
%Variance	37.468	20.884	16.473	8.8816	7.1392	4.7987	2.4965	1.8584
